# Structure and Intermolecular Interactions in Aqueous Solutions of Polyethylene Glycol

**DOI:** 10.3390/molecules27082573

**Published:** 2022-04-15

**Authors:** László Almásy, Oleksandr P. Artykulnyi, Viktor I. Petrenko, Oleksandr I. Ivankov, Leonid A. Bulavin, Minhao Yan, Vasil M. Haramus

**Affiliations:** 1 State Key Laboratory of Environment-Friendly Energy Materials, Southwest University of Science and Technology, Mianyang 621010, China; yanminhao@swust.edu.cn; 2 Institute for Energy Security and Environmental Safety, Centre for Energy Research, Konkoly-Thege Miklós út 29-33, 1121 Budapest, Hungary; 3 Faculty of Physics, Taras Shevchenko Kyiv National University, 01601 Kyiv, Ukraine; artykulnyi@gmail.com (O.P.A.); ivankov@jinr.ru (O.I.I.); bulavin221@gmail.com (L.A.B.); 4 BCMaterials, Basque Centre for Materials, Applications and Nanostructures, 48940 Leioa, Spain; viktor.petrenko@bcmaterials.net; 5 IKERBASQUE, Basque Foundation for Science, 48009 Bilbao, Spain; 6 Helmholtz-Zentrum Hereon, Max-Planck-Street 1, 21502 Geesthacht, Germany

**Keywords:** polymers in solutions, poly(ethylene glycol), micelle, Gaussian chain, small-angle neutron scattering

## Abstract

Aqueous solutions of polyethylene glycol are studied by small-angle neutron scattering over a broad range of polymer molecular masses and concentrations. The scattering data were modeled by a Gaussian chain form factor combined with random phase approximation, which provided good fits over the whole studied concentration range. The results showed that polyethylene glycol in the molecular mass range 0.4–20 kDa in water at physiological temperature T = 37 ${}^{{}^{\circ}}$C behaves like a random coil in nearly theta solvent conditions. The obtained results serve as a reference for the description of complex mixtures with PEG used in various applications.

## 1. Introduction

Polyethylene glycol (PEG) is the simplest water-soluble non-ionic synthetic polymer, having a broad range of applications from chemical technology to pharmaceutical and medical applications. It is used to cover the surfaces of colloidal particles in order to improve their biocompatibility, since the modified surface shows increased resistance to the adsorption of protein [1,2]. It is also used for covalent modification of biological macromolecules, peptides, liposomes, and other drug delivery systems [3,4,5]. Colloidal particles covered by PEG are not suppressed by the immune system, and their circulation time is increased up to 8–10 h, making them suitable for prolonged drug release [6,7,8]. Mixtures of PEG with anionic surfactants in aqueous solutions are used for various applications and were extensively studied during the last few years [9]. The solubility and colloidal properties of PEGylated particles depend strongly on the polymer molecular mass. Nanoparticles, oligomers, and low-molecular-weight compounds are usually covered with medium-sized polymers, with a mass of 20–50 kg/mol. Larger nanoparticles of sizes 50–100 nm are usually covered by low molecular weight PEG (3–10 kg/mol), since a further increase of the hydrodynamic radius leads to a reduction of the particle circulation lifetime [10,11]. Small nanoparticles can be covered by low-molecular-weight PEG (1.5–20 kg/mol), leading to an increase in the lifetime of magnetic nanoparticles introduced into the body [12,13]. PEG molecules having a size in the range of 2–8 kg/mol, at concentrations of 4–8 wt%, are used for the crystallization of proteins and biological macromolecules. In this process, the polymer occupies a large volume in the solution, displacing the protein. This induces the segregation and subsequent aggregation and formation of a solid crystalline phase [14,15]. Conformation of PEG (3.4 kg/mol) in salt solutions and the polymer partitioning into nanopores were investigated by small-angle scattering [16]. Recently, PEG in aqueous hybrid solvents was studied to reveal the influence of ionic liquids on the polymer behavior for industrial and medical applications [17]. For the ability to control all these properties, the behavior of the polymers in aqueous solutions in a broad range of conditions must be known.

The behavior of aqueous and non-aqueous PEG solutions is also an important topic of fundamental research in understanding polymer–solvent interactions. Light scattering, small-angle X-ray, and neutron scattering are the most efficient methods that provide structural information on the conformation and on the interactions of the polymer chains [18,19,20]. Various approaches can be used to interpret the scattering data and extract structural and thermodynamic information from the experiment. Scattering from polymer solutions can be modeled using empirical functions, which do not consider the polymer structure, but describe the density correlations in the solution at different length scales. Separate analysis of the different contributions can be subsequently related to the behavior of the polymers [19,20,21,22,23]. When analyzing the scattering data, assuming the explicit presence of the polymer, in the case of flexible polymers and small repeat units, the conformation of the polymer chains is modeled traditionally as flexible coils [24]. In dilute solutions, their conformation depends only on the solvent–polymer interactions. In semidilute solutions, the polymer–polymer interactions induce interference between scattering from the individual polymer chains and affect the chain conformations due to possible contacts with neighboring chains. These effects are often treated by the random phase approximation [24,25,26]. Furthermore, specific interactions can be included in these general models, such as sticky polymer–polymer attraction, which can explain some observations of the formation of large clusters [20,22,27]. Alternative models have also been reported recently, suggesting solid or crystalline-like PEG conformations in water, induced by specific solute–solvent interactions [28,29]. Scattering from swollen chemically cross-linked PEG gels follows more complicated models and generally shows a strong interference peak related to the characteristic distances and mesh size of the network [30].

The purpose of the present work was to study the structure and interaction of PEG molecules in an aqueous medium in a wide range of concentrations and molecular weights and provide a reference that can be further used in structural studies of PEG-containing systems. Previously, we investigated the structure of dilute aqueous solutions of PEG of molecular weights in the range of $M_{w}$ = 400–20,000 g/mol [31], as well as solutions of low-molecular-weight PEG over a broad concentration range [26]. In this paper, we used small-angle neutron scattering to analyze the polymer–polymer interactions via the scattering form and structure factor for polymer molecular weights 400–20,000 g/mol in the dilute and semidilute regions, at polymer mass fractions between 0.005 and 0.20.

The neutron scattering measurements were carried out at the physiological temperature of 37 ${}^{{}^{\circ}}$C, considering the specific purpose of utilizing PEG as the stabilizing agent in biocompatible magnetic fluids and drug delivery systems. Apart from describing the behavior and interactions in aqueous polymer solutions, our work is also related to studies of the interactions of polymers with surfactants [32,33,34] used in the preparation of water-based magnetic fluids [35,36,37].

## 2. Materials and Methods

PEG with nominal molecular weights $M_{w}$ = 400, 1000, 10,000, and 20,000 g/mol (PEG 0.4 kDa, 1 kDa, 10 kDa, and 20 kDa) were purchased from Sigma-Aldrich. They were dissolved in D${}_{2}$O (99.9 %) with a mass fraction of polymer in the range of 0.1–10 wt%, 0.6–20 wt%, 0.5–8 wt%, and 0.5–8 wt% for $M_{w}$ = 400, 1000, 10,000, and 20,000 g/mol, respectively. Solutions were prepared by weighing, stirred for a short time, and stored at room temperature during 2–4 days before measurements. Deuterated water was used to achieve a high contrast between the PEG and the solvent, as well as to reduce the incoherent background scattering coming from the hydrogen atoms of the solvent. The SANS experiment was conducted with the SANS-1 instrument at the research reactor FRG-1 [38] at Helmholtz-Zentrum Hereon in Geesthacht, Germany. A neutron beam with a mean wavelength of 8.1 Å and a full-width at half-maximum of 10% was obtained by a mechanical velocity selector. The distance between the sample and detector and the corresponding collimation length were varied from 0.7 to 9.7 m, providing a *q* range of 0.01–0.25 Å${}^{-1}$. The scattered neutrons were detected by a 2D position-sensitive ${}^{3}$He detector of 50 × 50 cm${}^{2}$ in size. The raw SANS spectra were corrected for the background from the solvent, sample cell, and other sources by conventional procedures. The two-dimensional isotropic scattering spectra were azimuthally averaged, converted to an absolute scale, and corrected for detector efficiency by dividing by the incoherent scattering of water [39], which was measured with a 1 mm path length quartz cell. Solutions were filled in Hellma quartz cells of a 1 mm light path and thermostated at a constant temperature of 37 ${}^{{}^{\circ}}$C. The differential scattering cross-section (hereinafter, the scattering intensity) was obtained as a function of the transmitted momentum modulus, q=(4π/λ)sin(θ/2), where λ is the incident neutron wavelength and θ is the scattering angle. The scattering intensity in an absolute scale (in units of cm${}^{-1}$) was obtained by using the scattering from a light water sample measured in a 1 mm path length quartz cell [39].

## 3. Results

**Figure 1:**
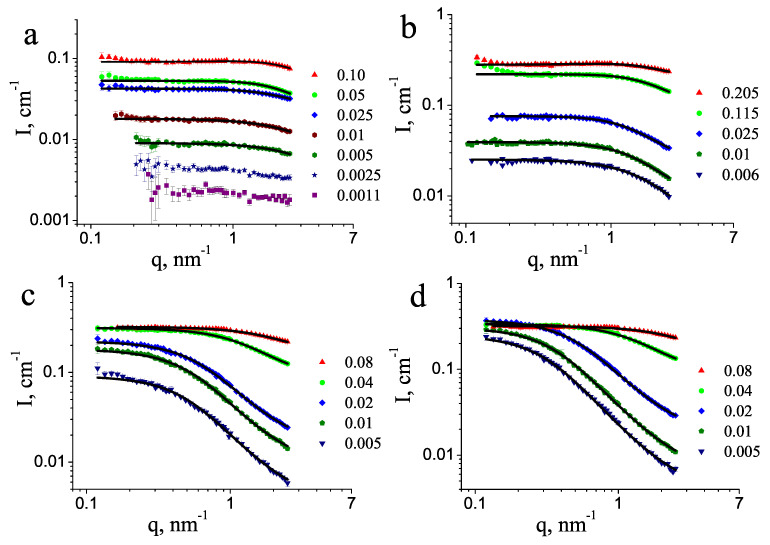
SANS curves for heavy water solutions of PEG of molecular mass (**a**) 0.4 kDa, (**b**) 1 kDa, (**c**) 10 kDa, and (**d**) 20 kDa. Solid lines show the model fitting according to Equations (1) and (2). The mass fractions of PEG are indicated.

Experimental SANS data for aqueous solutions of PEG 0.4 kDa, 1 kDa, 10 kDa, and 20 kDa are shown in Figure 1. At small polymer concentrations, the scattering curves correspond to the form factor of the polymer chains and are well approximated by the form factor of a Gaussian coil [23]. For large concentrations, the interference between neighboring polymer chains led to a decrease of the intensity in the low *q* range and the scattering data can be modeled by taking into account the structure factor.

As can be seen in Figure 1a,b, some increase in the scattering intensity occurred in the region of small *q* values (q<0.2 nm${}^{-1}$) for the high concentrations of 11.5 wt% and 20.5 wt%, which did not follow the smooth behavior characteristic of swollen polymer chains. This excess scattering can be related to the presence of a small amount of scattering objects, distinct from the polymer chains dissolved down to the molecular level. These objects are most likely clusters of polymer chains or undissolved polymer aggregates, making the studied samples two-phase systems. Because of their large size, only the tail of the scattering signal from the clusters was detected in the *q* range covered in the present experiment. Their minimal size can be estimated as ≈30 nm [26]. The equilibrium structure of the polymer solutions is reflected in the region of larger scattering angles, for *q* > 0.2 nm${}^{-1}$, and these data were used for the further analysis. We discuss the dilute and the concentration regions separately in the following sections.

### 3.1. Dilute Solutions

Traditionally, polymer chains in good solvents are understood to be dissolved down to the molecular level. In such a case, mathematical models can be constructed, the simplest and frequently used being the Debye formula describing the scattering intensity from non-interacting Gaussian coils. Such behavior is obeyed by polymer chains in theta solvent conditions, which in the case of PEG in water is reported to be around 50 ${}^{{}^{\circ}}$C [40,41]. At our conditions, the Gaussian coil behavior is seen to describe the scattering data well, shown in Figure 1. For the dilute PEG solutions, the measured scattering intensities are approximated by the Debye equation [42]:

(1) $$I\left(q\right)=I\left(0\right)P\left(q\right)S\left(q\right)+B=I\left(0\right)\frac{2\left[e^{-x}-\left(1-x\right)\right]}{x^{2}}+B,$$

where $x={\left(qR_{g}\right)}^{2}$, I(0) is the coherent forward scattering intensity, $R_{g}$ is the radius of gyration, *B* is the residual background, P(q) is the form factor of a Gaussian chain, and S(q) is the structure factor, which was close to 1 in the dilute solutions.

Three parameters were varied during the least-squares fitting to the experimental data, and the best fit values are collected in Table 1.

**Figure 2:**
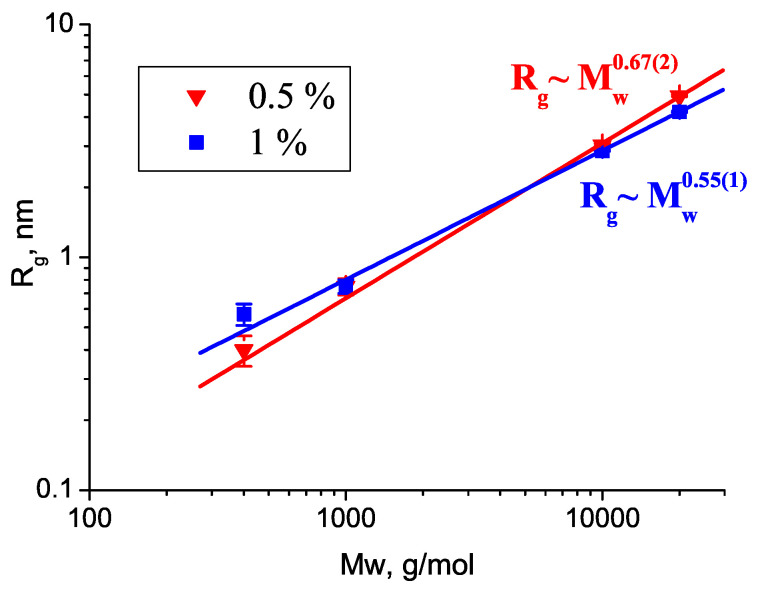
Dependence of the radii of gyration $R_{g}$ on the PEG molecular weight at different concentrations.

In the semidilute solutions, the apparent values of the gyration radii of PEG 1 kDa, 10 kDa, and 20 kDa decreased with increasing concentration (Table 1), indicating repulsive intermolecular interactions. The molecular weight dependence showed a power law $R_{g}$ ∝ $M_{w}^{P}$ (Figure 2). The exponent *P* decreased with increasing polymer concentration: P=0.67(2) and 0.55(1) for concentrations of 0.5 wt% and 1 wt%, respectively. These values were close to the scaling behavior of a random chain in a theta (P=0.5) or good solvent (P=0.588). The different values obtained for the two lowest concentrations showed that the polymer–solvent interactions did not match the theta conditions exactly, and also, the weak influence of the structure factor diminished the apparent polymer size as the concentration increased. For 0.5 wt% D${}_{2}$O solutions, with a PEG molecular weight of 2, 4, and 8 kDa, measured at T = 22 ${}^{{}^{\circ}}$C, the exponent value P=0.56 has been reported [28]. Molecular dynamics simulations of shorter PEG chains with polymerization degrees ranging from 9 to 36 resulted in P=0.51, showing that the polymer–water interactions were similar to that in a theta solvent [43]. More recent simulations using another force field gave P=0.48 for PEG with polymerization degrees 9–40 in water; however, in the water-miscible ionic liquid [bmim][BF${}_{4}$], the simulations resulted in very expanded polymer chains with P=0.9 [44]. In SANS experiments, however, a more realistic scaling with 0.55 was obtained, showing the good solvent nature of this ionic liquid [45].

In the dilute solutions, the radius of gyration can be related to the polymer chain length, by considering the non-interacting PEG molecules as ideal Gaussian coils, for which $R_{g}^{2}=Na^{2}/6$, where *N* ≈ 3.9, 9.9, 98.7, and 197.4 (for PEG 0.4 kDa, 1 kDa, 10 kDa, and 20 kDa, respectively) are the number of chain segments and a≈ 0.78 nm is the size of the segment, taken as the Kuhn length as determined by viscosimetry [46]. This gave the following estimated values for $R_{g}$: 0.63, 1.0, 3.2, and 4.5 nm for PEG 0.4 kDa, 1 kDa, 10 kDa, and 20 kDa, respectively, which were close to the experimentally found values of 0.4, 0.75, 3.0, and 4.9 nm for the lowest concentration of 0.5 wt%.

The experimental coherent forward scattering intensities, obtained by fitting the dilute solutions, were used to calculate the average molecular masses of the polymers, according to equation: $Mw_{exp}=\left(I\left(0\right)/S\left(0\right)\right)N_{a}/\left(\phi {\left(\Delta \rho \right)}^{2}V_{sp}\right)$, where ϕ is the polymer volume fraction, $V_{sp}$ is the specific volume of the polymer, and Δρ is the contrast of scattering length densities. The obtained values of molecular masses were as follows: 315, 880, 2950, and 10,060 g/mol for the four polymers. The experimental results for the samples with the lowest molecular mass, 400 Da and 1000 Da, were reasonably close to the nominal molecular mass, while they were lower by three- and two-times the nominal masses of the longer polymers, PEG 10 kDa and PEG 20 kDa. This difference can be attributed to the partial dissolution of the long polymers: the undissolved fraction did not contribute to the scattering intensity, modeled by the Gaussian chains and used to calculate I(0), but its trace can be noticed as the extra scattering of large clusters at *q* < 0.2 nm${}^{-1}$.

### 3.2. Concentrated Solutions

**Figure 3:**
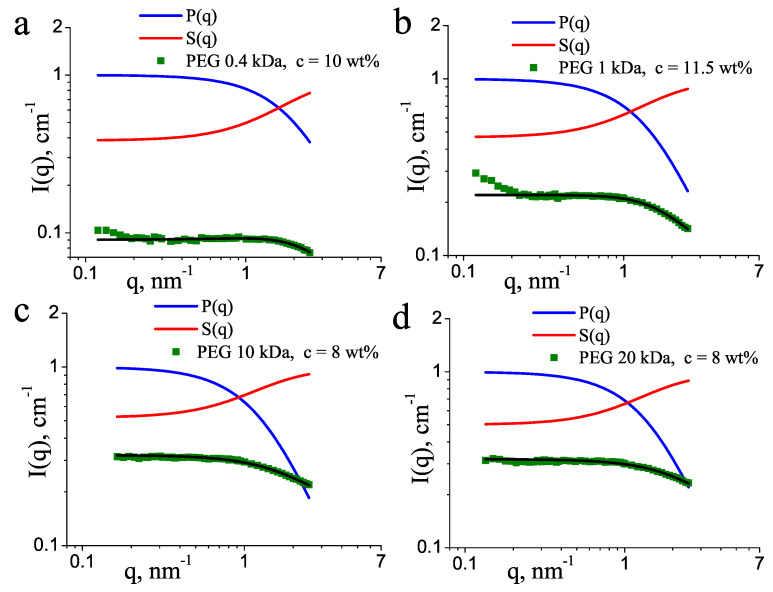
Form factors and structure factors for PEG of molecular mass (**a**) 0.4 kDa, (**b**) 1 kDa, (**c**) 10 kDa, and (**d**) 20 kDa. The concentrations of PEG are indicated. The experimental scattering intensities (symbols) and the model fits (black lines) are displayed in an absolute scale, whereas the form and structure factors are normalized as P(0)=1 and S(∞)=1.

For larger concentrations of PEG, above 2 wt%, the scattering data deviated from Equation (1) due to the increase of the interaction effects between the neighboring polymer chains. The simplest way to account for the interaction between the polymer coils is the use of the random phase approximation [47,48]:

(2) $$I\left(q\right)=I\left(0\right)\left(P\left(q\right)-\frac{A}{I(0)}P{\left(q\right)}^{2}\right)+B,$$

where P(q) is the Debye form factor, given by Equation (1), and parameter *A* is related to the second virial coefficient, which describes the effective monomer–monomer interaction. As can be seen in Figure 1, Equation (2) approximates well the experimental data at high polymer concentrations and the positive *A* values (Table 1) indicate repulsive monomer–monomer interactions, typical for good solvent conditions. The effect of the structure factor in the low-*q* part of the scattering curves is well seen in Figure 3, where the form and structure factors are shown separately, for the four molecular masses at similar concentrations of about 10 wt%. This depletion in the scattering intensity occurred in the broad *q* range 0–1 nm${}^{-1}$, with S(q) approaching a horizontal asymptote at *q* = 0. This structure factor was unrelated to the increase of the intensity in the lowest *q* < 0.2 nm${}^{-1}$. The latter effect seen in some of the scattering curves corresponds to scattering on large undissolved PEG aggregates and was not taken into account during the model fitting.

## 4. Discussion

The behavior of PEG in D${}_{2}$O at 37 ${}^{{}^{\circ}}$C, measured in the absence of any buffer salts, showed the characteristic picture of fully dissolved polymer chains, which in the dilute regime adopted the conformation of random Gaussian coils, and can be fit by the corresponding theoretical model equation using only three parameters. With increasing concentration, the inclusion of the structure factor becomes necessary, which in the present study was the random phase approximation. This approach allowed us to describe the scattering data of PEG solutions of low and medium molecular masses over a concentration range 0.5–20 wt%. Notably, these results showed that the solutions can be described as a two-phase system, consisting of random polymer chains dissolved on a molecular level and a certain amount of aggregated, undissolved molecules, these aggregates coexisting with the fully dissolved polymer chains. Such a coexistence of dissolved and non-dissolved polymers and surfactants were reported for both aqueous [27,49,50] and non-polar solutions [51]. Hammouda suggested [27] that this aggregation is of a hydrophobic character and happens due to local contacts between the ethylene groups, which are more frequent at higher concentrations. Since similar contacts may also occur intramolecular, they can lead to the deviation of the scattering curve from the Gaussian coil behavior. Model fitting does not allow distinguishing such effects, and their influence on the scattering can increase the uncertainty of the numerical values of the parameters obtained by the model analysis using the Debye formula, with or without the structure factor. Overall, their contribution is seen to be sufficiently small and having no effect on the observed shape of the scattering curves and their modeling by the mentioned form and structure factors. The important consequence of the partial dissolution of the polymer is, however, that the concentration of the dissolved polymer was noticeably lower than the total amount of polymer in the sample, especially for the high concentrations and for the longer polymers of 10 and 20 kDa.

The use of a Gaussian coil for the polymer chain form factor is based on the assumption that the theta condition is matched. For aqueous PEG solutions, different estimates of the theta temperature have been reported, ranging between 40 and 100 ${}^{{}^{\circ}}$C. In [23], Hammouda and Ho used a “model-free” form factor of a generic Lorentzian form, $I\left(0\right)/(1+{\left(qL\right)}^{m})$, and followed the asymptotic behavior of the scattering curve as a function of temperature. The *m* exponent varied monotonically in the interval 1.8–2.1 over the studied temperature range of 10–90 ${}^{{}^{\circ}}$C and was close to 2.0 (which corresponds to the high *q* behavior of a Gaussian chain) at temperatures 70–80 ${}^{{}^{\circ}}$C, indicating the effective theta condition. In our analysis, we used the Gaussian chain form factor with an asymptotic behavior of m=−2 at high *q* and obtained satisfactory agreement with the data for all conditions of the polymer masses and concentrations in salt-free heavy water solutions. Our results showed that SANS data can reasonably be described well by this approach, more generally than the scattering from a plate such as aggregates suggested for PEG 8 kDa in dilute ammonium sulfate buffer solutions [28]. It can also be noted that the solvent quality of H${}_{2}$O and D${}_{2}$O towards PEG is sensibly different and can be assessed by light scattering measurements [52]; however, neutron scattering studies for aqueous polymer solutions are almost exclusively performed with heavy water.

## 5. Conclusions

The structure of low- and medium-molecular-weight polyethylene glycols in aqueous solutions were studied at physiological temperature, 37 ${}^{{}^{\circ}}$C. Their behavior conformed to the behavior of random chains in good solvent conditions. The Gaussian chain form factor and random phase approximation described the conformation and the intermolecular interactions of the PEG polymer chains over a broad concentration range.

## Figures and Tables

**Table 1:** Parameters of Equations (1) and (2) obtained by fitting to SANS data of aqueous PEG solutions.

PEG Molecular Mass	PEG Mass Fraction	I(0), cm^−1^	$R_{g}$, nm	*B*, cm^−1^	A
400 g/mol	0.005	0.007(3)	0.4(1)	0.002	–
	0.01	0.01(1)	0.57(6)	0.006	–
	0.0	0.036(2)	0.89(1)	0.02	0.016(2)
	0.05	0.075(4)	0.85(6)	0.01	0.038(2)
	0.1	0.13(1)	0.80(6)	0.03	0.08(7)
1000 g/mol	0.006	0.025(1)	0.75(5)	0.002	–
	0.01	0.041(2)	0.75(4)	0.002	–
	0.025	0.076(1)	1.47(4)	0.02	0.06(1)
	0.115	0.30(1)	1.09(4)	0.06	0.16(1)
	0.205	0.26(1)	1.11(4)	0.12	0.16(1)
10,000 g/mol	0.005	0.07(2)	3.04(4)	0.003	–
	0.01	0.171(1)	2.87(2)	0.009	–
	0.02	0.207(1)	2.49(2)	0.01	–
	0.04	0.33(1)	1.49(3)	0.08	0.11(1)
	0.08	0.271(1)	1.24(3)	0.11	0.13(1)
20,000 g/mol	0.005	0.246(3)	4.94(5)	0.003	–
	0.01	0.302(3)	4.22(3)	0.006	–
	0.02	0.361(2)	2.99(2)	0.01	–
	0.04	0.39(1)	1.52(3)	0.10	0.15(1)
	0.08	0.260(5)	1.12(4)	0.12	0.13(1)

I(0): coherent forward scattering intensity; $R_{g}$: the radius of gyration, *B*: residual background; A: second virial coefficient.

## Data Availability

Not applicable.

## Abbreviations

The following abbreviations are used in this manuscript:

PEG	poly(ethylene) glycol
SANS	small-angle neutron scattering
$R_{g}$	radius of gyration
P(q)	form factor
S(q)	structure factor
